# William Lloyd Andriezen (c.1870–1906)

**DOI:** 10.1007/s00415-025-13534-x

**Published:** 2025-11-28

**Authors:** Andrew J. Larner

**Affiliations:** Department of Translational Neuroscience and Stroke, Institute of Neurology, University College London, London, UK

Histories documenting the discovery and characterization of the non-nervous cellular elements of the central nervous system generally begin with Rudolf Virchow’s concept of neuroglia (1850s) and then progress through Otto Deiters’ illustrations of stellate glial cells (1865), the work of Camillo Golgi (1870s) and Santiago Ramón y Cajal (1897, 1913) describing and illustrating glial cells, the discovery by Wilhelm His of the shared ectodermal origin of neurons and neuroglia (1889), Mihály von Lenhossék’s coinage of the name “astrocyte” (1895), and hence to Pío del Río-Hortega’s description of microglia and oligodendroglia (1919, 1921) and his distinction between fibrillar and protoplasmic astrocytes (1930s). A name absent from this chronology, but meriting inclusion for his work on the characterization of neuroglial cells and their alterations in certain pathological states, is William Lloyd Andriezen.

Of Sinhalese parentage, Andriezen received his medical and scientific training at University College London (UCL) between 1887 and 1893 (MB 1891; MD 1893). Of note, he won the Liston Gold Medal for Original Research in Pathology in 1891; he may have collaborated with the pioneer of neurosurgery at UCL, Victor Horsley (1857–1916), since a later publication mentioned findings in brain tissue resected from an epileptic patient by Horsley in 1891. Hence, Andriezen’s research activity was underway before he moved to the West Riding Asylum (WRA) in Wakefield, West Yorkshire, in January 1893 as Pathologist and Assistant Medical Officer (*BMJ* 1893;2:227). In 1894, he was elected to both the Neurological Society of London and the Medico-Psychological Association (forerunner of the Royal College of Psychiatrists). Thereafter he worked at the Metropolitan Asylum, Darenth, and at the Middlesex Hospital, London. He died at the age of 36, having been ill in the last year of his life [1, 2].

Andriezen’s two years in Yorkshire were highly productive of publications [3–6], although some of this research had been commenced at UCL [3, 5], indeed his seminal paper on neuroglia [3] was reprinted in the *Report of the Department of Pathology of University College, London. December, 1893* (pp. 1–7) presumably because his study began there in May 1892 (the volume was edited by Victor Horsley). Key to these various projects was the use of the Golgi silver chromate staining technique, his own modification of which Andriezen developed (described in [4] and *BMJ* 1894;1:909). This allowed him to study brain histology in both experimental animals and humans. He pursued a comparative and zoological method, his examinations spanning amphioxus, petromyzon (ammocoetes), fish, amphibians, kittens, rabbits, rats, monkeys, and humans. In the context of human pathological material, he has been credited with founding the world’s first “brain bank” for archiving over 100 brains at Wakefield [7].

**Fig. 1:**
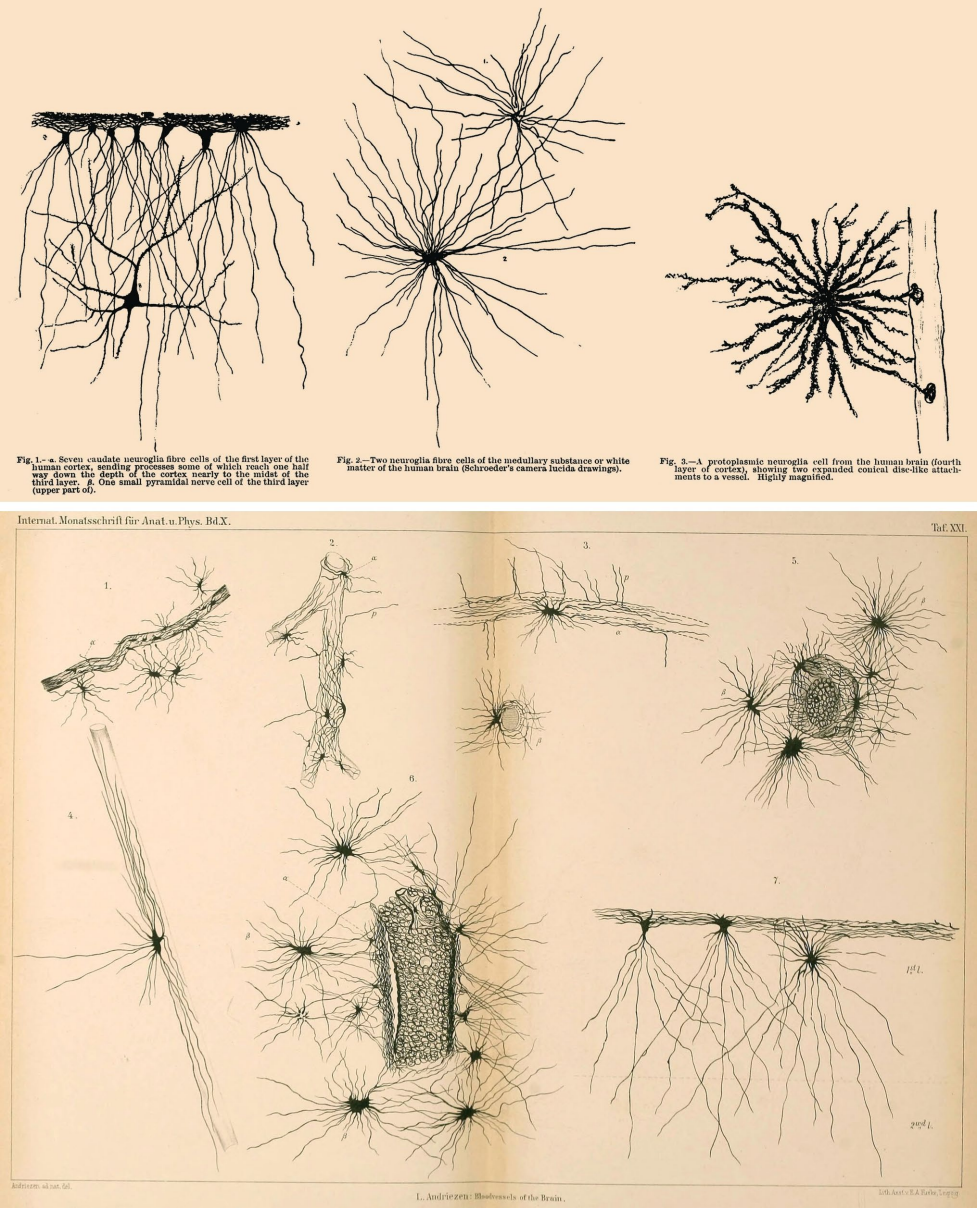
Andriezen’s drawings of various forms of glial cells, neuroglia fiber cell and the protoplasmic neuroglia cell, Golgi silver chromate stain, from [3] (top) and [4] (bottom)

Based initially on morphological grounds, Andriezen distinguished the “neuroglia fiber cell” [3] or “fiber elements” [4] from “the protoplasmic neuroglia cell” [3] or “protoplasmic cell elements” [4]. The latter were found to be abundant in gray matter and formed a perivascular feltwork supporting these elements and protecting nerve cells from damage (Fig. 1), whereas the fibrous forms were found especially in the white matter. Andriezen contrasted his subdivision of neuroglia with then existing views: “Hitherto all neuroglia cells in the adult brains have been included under one category of ‘spider’ cells (Deiters, Meynert, and others)” [3]. One of these parenthetical “others” was William Bevan-Lewis, the Medical Superintendent at WRA at this time. The omission of any mention of Andriezen in the second edition (1899) of Bevan-Lewis’s *Text-book of Mental Diseases* (“Thoroughly revised, enlarged, and in part re-written” according to the American edition), particularly his work on neuroglia, is perhaps surprising and suggests Bevan-Lewis may not have been willing to assimilate Andriezen’s findings, even though established in his own institution.

Using the Golgi staining method, Andriezen was also able to trace the development of nerve cells and cortical lamination, studies resulting in his *magnum opus* of nearly 150 pages published in *Brain* [6]. The title, “On some of the newer aspects of the pathology of insanity,” was in fact something of a misnomer, as only the concluding section examined pathological changes in a single cause of toxic insanity, namely alcoholic insanity. Here Andriezen illustrated the changes in neurons and protoplasmic neuroglia seen in this condition.

His other major research project, dating from his UCL years, related to the pituitary body which he concluded must have an “important trophic influence … on the central nervous system of vertebrates”, prompting him to predict the clinical consequences of ablation or destruction of the gland [5].

In addition to publishing, Andriezen was busy with presentations of his work: to the British Medical Association at Newcastle (August 1893); the Leeds and West Riding Medico-Chirurgical Society (February 1894); the Medico-Psychological Association at Dublin (June 1894); and the British Medical Association at Bristol (June 1894). The last of these addressed the then topical issue of insanity and “race decay”. He later presented on “The pathology of alcoholic insanity” at a meeting of the Neurological Society held in London in April 1895.

Further publications appeared following his departure from the West Riding Asylum [8, 9], some reporting cases seen at WRA with the affiliation “Late pathologist WRA” [8]. In “The pathogenesis of epileptic idiocy and epileptic imbecility”, both the macroscopic (convolutionary change) and microscopic findings in the brain were described, including cases shown at the Leeds and West Riding Medico-Chirurgical Society in April 1895 (*Lancet* 1895;1:1059–60). Andriezen noted “sclerotic overgrowth of the neuroglia fiber cells” in these brains, which we might now call “astrocytosis”, as well as destruction and atrophy of nerve cells and fibers, but provided no illustrations [8].

Andriezen published a series of articles “On the premature dementia of puberty and adolescence” in 1903, and “Melancholia as a form of insanity” and his ideas on “Insanity and race decay” in 1905, the latter apparently his final contribution. At the time of his premature death in 1906, it was noted that “His work in connection with neuropathology was known in every country” [1] and indeed his place in the history of glia, based particularly on his 1893 paper [3], has attracted some acknowledgements (e.g., [10]).
